# Supplementary material for: Effects of calcium-containing phosphate binders on cardiovascular events and mortality in predialysis CKD stage 5 patients
Source: PLoS One. 2020 Oct 30;15(10):e0241435. doi: 10.1371/journal.pone.0241435 (PMC7598463; doi:10.1371/journal.pone.0241435)
Supplement: Attachment — Submitted filename: Response to Reviewers.docx [file pone.0241435.s003.docx]

**Response to Reviewer:**

We thank you for your time and effort to improve this study. The manuscript has been revised according to your valuable comments, and the changes in the revised manuscript of the marked-up version have been highlighted in red font. The response to reviewer #2 was listed as below.

**Response to comments of Reviewer #2:**

**1. The authors have performed significant efforts to revise the manuscript based on the 4 reviewers' comments. One issue remained however inadequately addressed. The computation of daily elemental calcium received from phosphate binders would actually permit a more meaningful comparison of calcium exposure compared to ddd as it reflects a more accurate assessment of calcium exposure to patient and a more standardised unit of measure. There is probably no need to compare between different phosphate binders as highlighted by the authors if they were to compute these as the unit of measure would be standardised across patients.**

**Answer:** Thank you very much for your comment. We agreed with your comment that the computation of daily phosphate binders elemental calcium would permit more a meaningful comparison of calcium exposure compared to the defined daily dose because of a more accurate assessment of calcium exposure to the patient and a more standardized unit of measure. However, data in the National Health Insurance Research Database were de-identified by scrambling the identification codes of both patients and medical facilities, we could not track each patients’ accurate element calcium exposure or query the data at any level using this database. We added a section to describe this important limitation (line 301-303, page 18, marked-up version). To compare the effect of accumulated calcium content in CCPB on cardiovascular events, we used the defined daily dose (DDD) as a technical unit of measurement defined as the assumed usual maintenance dose per day for a drug used for its main indication in adults.
